# The Use of Acellular Fish Skin Grafts in Burn Wound Management—A Systematic Review

**DOI:** 10.3390/medicina58070912

**Published:** 2022-07-09

**Authors:** Hanna Luze, Sebastian Philipp Nischwitz, Christian Smolle, Robert Zrim, Lars-Peter Kamolz

**Affiliations:** 1 Division of Plastic, Aesthetic and Reconstructive Surgery, Department of Surgery, Medical University of Graz, 8036 Graz, Austria; sebastian.nischwitz@medunigraz.at (S.P.N.); christian.smolle@medunigraz.at (C.S.); dr.zrim@me.com (R.Z.); lars.kamolz@medunigraz.at (L.-P.K.); 2 Research Unit for Tissue Regeneration, Repair and Reconstruction c/o Division of Plastic, Aesthetic and Reconstructive Surgery, Department of Surgery, Medical University of Graz, 8036 Graz, Austria; 3 International University of Monaco, 98000 Monaco-Ville, Monaco; 4 COREMED—Cooperative Centre for Regenerative Medicine, Joanneum Research Forschungsgesellschaft mbH, 8010 Graz, Austria; 5 Research Unit for Safety in Health c/o Division of Plastic, Aesthetic and Reconstructive Surgery, Department of Surgery, Medical University of Graz, 8036 Graz, Austria

**Keywords:** acellular fish skin, fish skin grafts, burn injury, burn wound management

## Abstract

*Background and Objectives:* Burn wound healing and management continues to be a major challenge for patients and health care providers resulting in a considerable socio-economic burden. Recent advances in the development of applicable xenografts as an alternative to split-thickness skin grafts have allowed for the development of acellular fish skin. Acellular fish skin acts as a skin substitute, reducing inflammatory responses and advancing proinflammatory cytokines that promote wound healing. Due to these beneficial wound healing properties, acellular fish skin might represent an effective treatment approach in burn wound management. *Materials and Methods:* A systematic review of the literature, up to March 2022, was conducted using the electronic databases PubMed and Web of Science. Titles and abstracts were screened for the following key terms (variably combined): “fish skin”, “fish skin grafts”, “acellular fish skin”, “Omega3 Wound matrix”, “xenograft”, “burn injury”, “burns”. *Results:* In total, 14 trials investigating the effects of acellular fish skin in burn wounds or split-thickness donor sites were determined eligible and included in the present review. Existing evidence on the use of acellular fish skin indicates an acceleration of wound healing, reduction in pain and necessary dressing changes as well as treatment-related costs and improved aesthetic and functional outcomes compared to conventional treatment options. *Conclusions:* Acellular fish skin xenografts may represent an effective, low-cost alternative in treatment of superficial- and partial-thickness burns. However, results mainly originate from preclinical and small cohort studies. Future larger cohort studies are warranted to elucidate the full potential of this promising approach.

## 1. Introduction

Burn wound healing and management as a complex and long-lasting process continues to represent a major challenge for patients and health care providers resulting in considerable socio-economic burdens [1,2]. In deep dermal and full-thickness burn injuries, early excision and application of split-thickness skin grafts is the established main treatment option to achieve early wound closure and avoid common complications such as sepsis, multi-organ failure and acute kidney injury [2,3]. However, split-thickness skin grafting may not be possible, e.g., in extended burns with limited donor skin availability [4]. Even if available, outcomes may be suboptimal, including significant donor site morbidity [4]. Subsequently, there is a great demand for treatment options capable of achieving early and complete wound coverage while retaining normal skin function [4].

A variety of dressings are currently available for superficial partial-thickness burns, such as silver-impregnated, alginate, hydrocolloid, hydrogel, silicone-coated nylon, polyurethane film or biosynthetic dressings, without a gold standard being defined [5]. The main components of a complication-free treatment to be targeted via dressing application are improvement and acceleration of the wound healing process, prevention of potential colonization with pathogenic biofilms, or the reduction of necessary dressing changes [2,6].

In order to assist with the progression of the wound healing phases, numerous biologic materials such as allo- or xenografts have evolved [7]. A recent advance in the development of applicable xenografts was acellular fish skin (AFS) grafts harvested from two major species such as the Nile Tilapia (*Oreochromis niloticus*) or the North Atlantic cod (*Gadus morhua*) (Kerecis^®^ Omega3, Kerecis, Isafjordur, Iceland). AFS of the Nile Tilapia originates from the Nile basin in East Africa and is usually provided by local fish farms [8]. While Nile Tilapia AFS can exclusively be used in tropical or subtropical regions of the world [8], Kerecis^®^ Omega3 dressings of the North Atlantic cod are available worldwide and were initially approved for the treatment of various wounds by the US Food and Drug Administration in 2013 [5,7].

AFS contains collagen, fibrin, proteoglycans and glycosaminoglycans, therefore acting as a skin substitute [9,10]. In comparison to mammalian acellular grafts (e.g., porcine, bovine), AFS grafts do not carry a risk of disease transmission such as bovine spongiform encephalopathy and variant Creutzfeldt-Jakob disease [11], and can therefore undergo a simpler sterilization process retaining an omega-3 fat source [12]. Previous evidence states that omega-3 polyunsaturated fatty acids, eicosapentaenoic acid and docosahexaenoic acid, which are abundant in fish skin, reduce inflammatory responses and advance proinflammatory cytokines that promote wound healing [9]. AFS containing these omega-3 polyunsaturated fatty acids can therefore facilitate the transition out of the inflammatory phase of the wound healing process [7].

Due to these beneficial wound healing properties, Omega-3-rich AFS may be used in a broad spectrum of applications: Recent studies report good clinical outcomes after application in, e.g., chronic diabetic foot ulcers [13], calciphylaxis wounds [14], necrotic angiodermatitis [15], iatrogenic calcinosis cutis [16] or even for neovaginoplasty in patients with Mayer-Rokitansky-Küster-Hauser syndrome [17]. AFS may also present an effective treatment option in burn wound management, since studies indicate accelerated wound healing, pain reduction, decrease in necessary dressing changes as well as treatment-related costs [5].

## 2. Methods

### 2.1. Aim Construct

This review aims at summarizing the existing evidence published in peer-reviewed journals on the use of AFS in the treatment of burn injuries.

### 2.2. Literature Search

We reviewed the medical literature in order to identify all in vivo studies investigating the use of AFS in burn injuries. A systematic search of the literature published until March 2022 was conducted using the electronic databases PubMed and Web of Science as well as relevant reference lists. The search strategy included the following terms (variably combined): “fish skin”, “fish skin grafts”, “acellular fish skin”, “Omega3 Wound matrix”, “xenograft”, “burn injury”, “burns”. Additionally, the reference lists of included articles were manually screened for further relevant publications.

**Figure 1:**
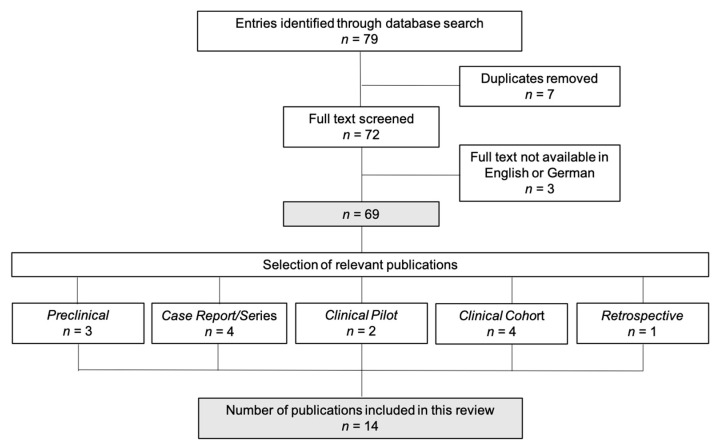
Study inclusion. In total, 79 publications evaluating the use of acellular fish skin grafts in burn injuries were identified. After elimination of duplicates (*n* = 7), full-text evaluation of the remaining publications was performed (*n* = 72). Only articles published in English or German language were included (exclusion of *n* = 3). 14 publications were determined eligible and included in the present review. Study types were defined as follows: preclinical study (*n* = 3), case report/series (*n* = 4), clinical pilot study (*n* = 2), clinical cohort study (*n* = 4), retrospective data analysis (*n* = 1).

In total, 79 human and animal in vivo publications evaluating the use of acellular fish skin grafts in burn injuries were identified. If the abstract did not determine eligibility, full-text evaluation was performed. Human or animal in vivo investigation of the effects of AFS in burn wounds or split-thickness donor sites was determined as the fundamental inclusion criterion. After elimination of duplicates (*n* = 7), full-text evaluation of the remaining publications was performed as shown in Figure 1. All results were independently screened and assessed by two researchers to identify any relevant studies for inclusion and the final set was agreed upon by serial discussion/assessment rounds for any discrepancy in selection. Cohen’s kappa coefficient was calculated at each stage of title, abstract and full text review to ascertain inter-rater variability between the two reviewers. Only articles published in English or German were included (exclusion of *n* = 3).

### 2.3. Outcome Data

Studies were eligible for inclusion if they reported any of the following outcome measures:

Primary Outcomes:

- Reepithelialization time.
- Change of the wound surface area over time.
- Secondary outcomes:
- Number of dressing changes,
- Cost of the dressings,
- Level of pain associated with the application, change or removal of the dressing,
- Analgesic intake,
- Hospital length of stay,
- Need for further intervention (surgery),
- Scar quality.

### 2.4. Reporting of the Systematic Review and Evidence-Based Process

PRISMA guidelines were applied to report the systematic review and evidence-based process (https://doi.org/10.1371/journal.pmed.1000097 (accessed on 27 April 2022)) The retrieval process reported different a variety of study types as well as the utilization of two main AFS grafts. Due to the novel nature of this topic in burn wound management, the guidelines required adaption. To ensure comparability, rigorous critical appraisal was applied to determine the quality of the studies retrieved and ensure inclusion of comparable, valid and relevant evidence.

## 3. Results and Discussion

In total, 14 publications investigating the effects of AFS in burn wounds or split-thickness donor sites were determined eligible and included in the present review. Agreement between reviewers was high with Cohen’s kappa coefficient calculated as 0.75, 0.81 and 0.94 for the title, abstract and full-text review stages, respectively. All trials included were published in English and study types were defined as follows: preclinical study (*n* = 3), case report (*n* = 3), case series (*n* = 1), clinical pilot study (*n* = 2), clinical cohort study (*n* = 4), retrospective data analysis (*n* = 1). The effects of Nile Tilapia AFS were evaluated in seven trials whereas Kerecis^®^ Omega3 dressings were used in five trials. A single preclinical trial evaluated AFS obtained from grass carps [18]. Study details are depicted in Table 1 and Table 2.

No minor or major adverse events or drop-outs were reported in trials included in the present review. Furthermore, follow-up period was only determined by Costa et al. (1 week) [19] and Wallner et al. (12 months) [20].

Included trials primarily focus on the total reepithelialization time of burn wounds treated with AFS as well as a potential reduction of pain, necessary dressing changes and treatment-related costs. Furthermore, long term outcomes as well as a possible application of AFS in split-thickness donor sites were evaluated. Since not all studies included control groups or comparison products, no general statement regarding the level of statistical significance can be given. When control groups or other dressings were included and evaluated, two-sided *p*-values < 0.05 were considered statistically significant.

Promising results supporting the use of AFS in the treatment of burn injuries were retrieved from trials existing to date and will be described in detail within the following chapters.

### 3.1. Reepithelialization Time

The total reepithelialization time of burn wounds treated with AFS represents the main focus in trials published to date. In already established standard treatment options, reepithelialization of superficial partial-thickness burn (SPTB) wounds is usually expected within 2 weeks and may take more than 3 weeks for deep partial-thickness burn (DPTB) wounds [21,22]. Preclinical burn-injury studies in in rabbits [18,23] and pigs [4] indicated a potential acceleration of wound healing in burn wounds treated with AFS already. These preclinical assumptions were recently studied in various human in vivo trials [5,20,24].

Similar to prior results [21,22], a phase II randomized, controlled study by Lima Júnior et al. reports a total reepithelialization time of approximately 10–11 days in SPTB and approximately 21 days in DPTB with the conventional treatment option of silver sulfadiazine cream 1% [25], a burn cream providing broad antibacterial activity, forming a temporary barrier and promoting reepithelialization [26]. A reduced period of time until complete reepithelialization was achieved using Nile Tilapia AFS grafts: an average reduction of 1.43 days for outpatients and 1.14 days for inpatients in comparison to conventional treatment options was observed [25]. Accelerated wound healing was also reported in the following phase III trial including 115 patients with SPTB [5]. The average total reepithelialization time was reduced by 0.5 days by treatment with Nile Tilapia AFS compared to the conventional treatment option with silver sulfadiazine cream 1% [5]. Evidence suggests that AFS may also be a suitable option for SPTB wound treatment in children, where total reepithelialization time of 10 days was achieved in a 3-year-old boy with a SPTB of 18% the total burn surface area (TBSA) [19].

In comparison to SPTB, the reepithelialization of DPTB can be challenging and prolonged, hence effective treatment options are warranted if split-thickness skin grafting cannot be per-formed or must be delayed [21,22]. Promising data on the use of Kerecis^®^ Omega3 in DPTB were found in a preclinical study in six female Yorkshire pigs evaluating the total reepithelialization time of DPTB and full-thickness burn wounds [4]. The authors reported faster reepithelialization and reduction of the wound size in wounds treated with AFS compared to fetal bovine dermis (Primatrix ^TM^, Integra LifeSciences, Princeton, NJ, USA) [4].

Despite an appropriate study design and quality of preclinical studies included, results may not fully be convertible into clinics. However, accelerated wound healing of DPTB is also reported in human in vivo studies: A case report of a 23-year-old male patient with mixed dermal burn wounds reports a total reepithelialization time of 17 days after Nile Tilapia AFS grafting [21]. In a retrospective case–control study by Wallner et al., a significantly faster reepithelialization time of 22 ± 6.3 days was observed in mixed dermal burn wounds treated with Kerecis^®^ Omega3 following enzymatic debridement (NexoBridTM, MediWound Germany GmbH, Rüsselsheim, Germany) compared to the treatment combination of enzymatic debridement and Suprathel^®^ (PolyMedics Innovations GmbH, Denkendorf, Germany) (45.6 ± 6.6 days) or split-thickness skin grafting (34.7 ± 12.5 days) [20]. According to these findings, treatment with AFS results in at least equal, but potential accelerated wound healing compared to conventional approaches, possibly benefitting other parameters such as necessary dressing changes or treatment-related costs. However, comparability of different substitutions may be limited due to their indication based on burn depth [20]. Ultimately, larger, randomized cohort studies are necessary to further elucidate these findings.

### 3.2. Pain Intensity

In a global survey on properties of an ideal burn wound dressing, pain reduction as well as pain-free dressing changes ranked second [6], highlighting the demand for this characteristic in burn wound management. Existing evidence to date reports promising effects of AFS in terms of pain reduction: A phase II randomized, controlled study by Lima Júnior et al. revealed a reduced overall pain intensity evaluated by the visual analogue scale and a decreased need for anesthetics in patients treated with Nile Tilapia AFS compared to the conventional treatment with silver sulfadiazine cream 1% [25]. Similar results were found in the following phase III trial, with additional reports of a decreased score in Burn Specific Pain Anxiety Scale (BSPAS) and mechanical pain threshold measurements using Electronic von Frey “Digital Analgesimeter” (Insight Eq-uipamentos Ltd.a., São Paulo, Brazil) [5].

Wallner et al. reported decreased pain and itch as expressed as Patient and Observer Scar As-sessment Scale (POSAS) scores in wounds with a combined treatment of enzymatic debridement and Kerecis^®^ Omega3 compared to Suprathel^®^ as an absorbable, synthetic skin substitute or split-thickness skin grafts [20].

In pediatric burn wound management, the total amount of analgesics required and the pain throughout the treatment is equal when compared to conventional treatment options [24]. These consistently obtained results on a potential pain reduction support a clinical application of AFS in selected cases. Moreover, a reduction of pain and discomfort, enabled through this novel approach, is associated with improved health and functioning in long-term outcomes [27].

### 3.3. Dressing Changes

The requirement of fewer (pain-free) dressing changes in burn wound management is of fundamental interest [6] and might be feasible with this novel approach. Most studies report primary dressing changes only, if the AFS does not adhere to the wound bed properly [24]. Despite a broad application spectrum of AFS, attachment may be difficult in areas with skin folds (e.g., face, neck), hence other treatment options might be preferable [19].

When compared to conventional treatment options, Nile Tilapia AFS led to fewer dressing changes (average reduction of 3.72 for outpatients and 8.67 for inpatients) in SPTB [17]. Fewer dressing changes may have been required due to good adherence of the biomaterial to the wound bed [21] and may be of particular interest in pediatric burn treatment. A recent phase II randomized pilot study in 30 children reported a reduced total number of dressings in the Nile Tilapia AFS group (3.00 ± 0.76) when compared to the silver sulfadiazine cream 1% group (9.27 ± 1.39) [24]. The reduction of necessary dressing changes and therefore inpatient treatment days can be associated with a reduction of treatment-related costs [28] and simultaneously increase the patients’ comfort [6].

### 3.4. Treatment-Related Costs

Modern burn care is perceived as an expensive, resource-intensive endeavor, requiring specialized equipment, medical personnel and facilities to provide optimal care [28]. Studies on treatment-related costs are scarce; however, the establishment of effective, low-cost treatment options is of utmost importance, particularly for low- and middle-income countries, where over 85% of burn injuries are registered [28].

A phase III randomized, controlled study by Lima Júnior et al. targeted costs related to the treatment of SPTB with Nile Tilapia AFS [5]. A reduction of the final treatment related costs per patient by 42.1% compared to other treatment options was shown [5]. The authors furthermore report a mean cost of USD 11 (±1USD) per patient when treated with Nile Tilapia AFS, compared with USD 19 (±1USD) when treated with silver sulfadiazine cream 1% [5]. Special preparation and storage techniques of AFS might even extend storage of sterile tissue and decrease costs related to distribution and transport [29].

Due to limited financial resources, the use of AFS as a cost-effective alternative to modern synthetic and biosynthetic dressings might benefit public health systems of developing countries [5]. However, cost-effectiveness studies exclusively exist on the geographically limited Nile Tilapia AFS [5].

Future studies on the globally available, but high-cost AFS dressing Kerecis^®^ Omega3 [30] are of utmost importance to evaluate a potential benefit on strained health care systems worldwide.

### 3.5. Application in Split-Thickness Donor Sites

Current strategies not only aim at improving of burn wound healing and outcome, but also at reducing required split-thickness skin grafting [4]. If split-thickness skin grafting is performed, AFS might be used as a biocompatible dressing option: Yoon et al. investigated the wound healing properties of Kerecis^®^ Omega3 in split-thickness donor sites of burn patients in comparison to a bovine collagen skin graft (ProHeal^®^ Collagen Wound Dressing, MedSkin Solutions, Billerbeck, Germany) [31]. Treatment with Kerecis^®^ Omega3 demonstrated accelerated wound healing (9.1 ± 1 days) compared to the treatment with ProHeal^®^ (10.7 ± 1.45 days) or untreated sites by nearly two days [31]. As suggested in prior studies, wound healing acceleration might be caused due to an increased cell proliferation and a synergistic effect of the biophysical properties of AFS [31]. In a different case series by Alam et al., wound healing of split-thickness donor sites treated with Kerecis^®^ Omega3 showed an average total reepithelialization time of 11.5 days (range 10–16 days) [3], whereby longer reepithelialization time is observed in larger donor sites or thicker split-thickness grafts [30].

Despite the authors evaluated wound healing abilities of Kerecis^®^ Omega3 in split-thickness donor sites due to their comparability with SPTB, the application in split-thickness donor sites might also be a viable treatment alternative with high therapeutic potential.

### 3.6. Long-Term Outcomes

Burn injuries are often associated with persisting complications related to scarring—such as contractures, weakness, changes in thermoregulation, itching and pain often impairing the patients’ body image and psychosocial wellbeing [27]. Long-term results of burn wound treatment with AFS are limited to one retrospective case–control study by Wallner et al., who demonstrated a significantly superior elasticity in regenerated skin treated with a combination of enzymatic debridement and Kerecis^®^ Omega3 compared with combined treatment with Suprathel^®^ 12 months postoperative [20].

Furthermore, a higher sebum and water content compared to wounds treated with Suprathel^®^ or split-thickness skin grafts was observed [20]. The relative water content is comparable to physiological skin and is associated with improved functional and aesthetic outcomes regarding elasticity, skin thickness and pigmentation [20]. Patients with risk factors for pathological wound healing and scarring—such as a large DPTB surface area, prolonged wound healing, multiple surgeries [32]—without the possibility of split-thickness skin grafting may particularly benefit from AFS application in the long term.

### 3.7. Limitations

The relevance of existing trials to date, either preclinical or clinical, is limited due to a small size of study cohorts and results cannot be pooled. Furthermore, studies on the Nile Tilapia AFS are mainly performed by the same group and findings are geographically limited due to the regional availability of Nile Tilapia. These findings might be transferrable to a treatment with Kerecis^®^ Omega3, however future clinical (comparison) trials with a sufficient number of subjects are warranted to elucidate the full potential of this promising approach. Furthermore, comparability of different substitutions may be limited due to their general use (e.g., silver sulfadiazine cream 1%) or their indication based on burn depth. Finally, our review is limited to articles retrieved from PubMed and Web of Science only with a slight possibility of missing other trials.

## 4. Conclusions

Despite being considered as the current gold standard for early wound closure in deep dermal and full-thickness burn injuries, split-thickness skin grafting may be limited in selected cases [31]. Current strategies aiming at improvement of burn wound healing and outcome as well as the reduction of necessary split-thickness skin grafting mainly include biological and synthetic skin replacement products [4]. The novel approach of AFS xenografts may represent an effective, low-cost alternative for the treatment of DPTB as well as SPTB since existing evidence indicates accelerated wound healing, reduction of pain and necessary dressing changes as well as improved long-term outcomes. Future large-cohort studies are of utmost importance to elucidate the full potential of this promising approach.

## Figures and Tables

**Table 1:** Preclinical studies evaluating the use of acellular fish skin in burn wounds.

Study	Acellular Fish Skin	Comparison Product	Animal Model	Scalding Conditions	Treatment Period	Endpoints	Main Findings
Accelerated Wound Closure of Deep Partial Thickness Burns with Acellular Fish Skin Graft. (Stone II et al., 2021) [2]	North Atlantic cod (Kerecis^®^ Omega3)	Fetal bovine dermis (Primatrix ^TM^)	6 female Yorkshire pigs of weights 51.8 ± 3.3 kg at the time of the burn wound creation	Creation of ten 5 cm × 5 cm wounds (4 DPT and 6 FT) with a thermocouple brass burn device by Alam et al. [3] heated to 100 °C. - Applied pressure: 4000 g/cm^2^ - Contact time: DPT: 25 s FT: 30 s	60 days	Reepithelialization time, skin function (skin moisture properties, microcirculation)	AFS: faster reepithelialization time in DPT and FT wounds.
A comparative study of two porous sponge scaffolds prepared by collagen derived from porcine skin and fish scales as burn wound dressings in a rabbit model. (Shi et al., 2020) [4]	Grass carp	Porcine skin-derived Collagen, dry gauze, Vaseline gauze	2 New Zealand white rabbits	Creation of ten 2 cm × 2 cm wounds with 35 layers of boiled gauze which was applied with gravitational pressure. - Contact time: 20 s	28 days	Wound size, dressing properties	AFS: faster wound healing after 12 days, higher moisture permeability
Marine Collagen Peptides from the skin of Nile Tilapia (*Oreochromis* *niloticus*): Characterization and Wound Healing Evaluation. (Hu et al., 2017) [5]	Nile Tilapia	-	48 New Zealand white rabbits	Creation of one DPT 4 × 4 cm burn wound with a scalding device (YLS-5Q, Bejing, China) heated to 100 °C. - Applied pressure: 1000 g - Contact time: 5 s	18 days	Reepithelialization time, histological analysis of the skin structure integrity, cell types and granulation tissue	AFS: faster reepithelialization time in comparison to the control group. Reduction of inflammation and promotion of granulation tissue formation

Abbreviations: acellular fish skin (AFS); deep partial-thickness (DPT); full thickness (FT), seconds (s).

**Table 2:** Clinical studies evaluating the use of acellular fish skin in burn wounds and split-thickness donor sites.

Study	Study Type	Fish Skin	Comparison to	Study Cohort	Treatment Period	Endpoints	Main Findings
The Use of Intact Fish Skin as a Novel Treatment Method for Deep Dermal Burns following Enzymatic Debridement: A retrospective Case-Control Study. (Wallner et al., 2022) [6]	Retrospective case–control study	North Atlantic cod (Kerecis^®^ Omega3)	absorbable, synthetic skin substitute (Suprathel^®^) in SPTB split-thickness skin graft in DPTB	12 patients (age range 18–60 years) with SPTB or DPTB mean TBSA of 12.5 ± 9.4% after enzymatic debridement	28 days	Wound quality assessment and size, reepithelialization time, scar quality	AFS: accelerated wound healing (total reepithelialization time of 22 ± 6.3 days), higher water-storage capacity, improved aesthetic and functional outcomes, decreased pain and itching
Wound healing ability of acellular fish skin and bovine collagen grafts for split-thickness donor sites in burn patients: Characterization of acellular grafts and clinical application. (Yoon et al., 2022) [7]	In vitro and clinical comparison study	North Atlantic cod (Kerecis^®^ Omega3)	Bovine collagen graft (ProHeal^®^)	52 patients with acute burns who underwent split-thickness skin grafting	Up to 17 days	In vitro: cellular responses to the grafts In vivo: reepithelialization time, wound complications	AFS: accelerated reepithelialization time by 2 days
Nile Tilapia Fish Skin-Based Wound Dressing Improves Pain and Treatment-Related Costs of Superficial Partial-Thickness Burns: A Phase III Randomized Controlled Trial. (Lima Júnior et al., 2021) [8]	Open-label, monocentric, Phase III Randomized Controlled Trial	Nile Tilapia	Silver Sulfadiazine Cream 1%	115 patients (age range: 18–70 years) with SPTB < 15% TBSA	Up to 11 days	Reepithelialization time, number of dressing changes, treatment related costs, pain intensity	AFS: Fewer days of reepithelialization and dressing changes. Lower analgesic needs and scores in BSPAS and mechanical pain threshold measurements. Reduction of average treatment related costs per patient by 42.1%
A Randomized Comparison Study of Lyophilized Nile Tilapia Skin and Silver-Impregnated Sodium Carboxymethylcellulose for the Treatment of Superficial Partial-Thickness Burns. (Lima Júnior et al., 2021) [9]	Open-label, randomized, prospective, controlled pilot study	Nile Tilapia	silver-impregnated sodium carboxymethylcellulose dressing (Aquacel Ag^®^)	24 patients (age range 18–70 years) with SPTB ≤ 10% TBSA	Up to 11 days	Number of dressing changes, pain intensity, pain-related anxiety, analgesic intake	AFS: Reduced number of dressing changes, lower overall pain intensity measured via VAS score. Comparable analgesic intake and pain-related anxiety.
Lyophilised tilapia skin as a xenograft for superficial partial thickness burns: a novel preparation and storage technique. (Lima Júnior et al., 2020) [10]	Case Report	Nile Tilapia	-	33-year-old female patient with SPTB of 10% TBSA	10 days	Reepithelialization time	Good adherence to the wound bed, total reepithelialization time of 10 days
Innovative Burn Treatment Using Tilapia Skin as a Xenograft: A Phase II Randomized Controlled Trial. (Lima Júnior et al., 2020) [11]	Open-label, monocentric, Phase II Randomized Controlled Trial	Nile Tilapia	Silver Sulfadiazine Cream 1%	62 patients (age range: 18–50 years) with SPTB ≤ 20% TBSA or DPTB between 5 and 15% TBSA	Up to 23 days	Reepithelialization time, number of dressing changes, burn improvement, anesthetic/analgesic intake, pain intensity	AFS: Fewer days of reepithelialization and dressing changes. Lower pain intensity and amount of anesthetics/analgesics
Innovative treatment using tilapia skin as a xenograft for partial thickness burns after a gunpowder explosion. (Lima Júnior et al., 2019) [12]	Case Report	Nile Tilapia		23-year-old male patient with 16% TBSA SPTB and DPTB	Up to 17 days	Reepithelialization time	Good adherence of the biomaterial to the wound bed, reepithelialization of SPTB in 12 days and 17 days in DPTB
Pediatric Burn Treatment Using Tilapia Skin as a Xenograft for Superficial Partial Thickness Wounds: A Pilot Study. (Lima Júnior et al., 2019) [13]	Open-label, monocentric, randomized phase II pilot study	Nile Tilapia	Silver Sulfadiazine Cream 1%	30 children (age range: 2–12 years) with SPTB	Up to 11 days	Reepithelialization time, number of dressing changes	AFS: Reduced total number of dressings (3.00 ± 0.76) in comparison to the Silver Sulfadiazine cream 1% group (9.27 ± 1.39) Comparable reepithelialization time and rate, anesthetic and analgesics intake
Use of Tilapia Skin as a Xenograft for Pediatric Burn Treatment: A Case Report. (Costa et al., 2019) [14]	Case Report	Nile Tilapia		3-year-old boy with SPTB of 18% TBSA	10 days	Reepithelialization time	Total reepithelialization time of 10 days
Acellular Fish Skin Grafts for Management of Split Thickness Donor Sites and Partial Thickness Burns: A Case Series. (Alam et al., 2019) [2]	Case series	North Atlantic cod (Kerecis^®^ Omega3)		10 patients (age range 18–90 years) undergoing split-thickness skin grafting for burn injuries	Up to 16 days	Reepithelialization time, pain	Total reepithelialization time of 11.5 days (range: 10–16), Average pain score of 2.3 (range 1–4) of 10 at dressing changes
Acellular fish skin matrix on thin-skin graft donor sites: a preliminary study. (Badois et al., 2019) [15]	Prospective, comparative, before-after cohort study	North Atlantic cod (Kerecis^®^ Omega3)	Paraffin gauze (Jelonet)	21 patients (age range: 33–84 years) with split-thickness skin graft donor sites of 30–45 cm^2^	Up to 134 days	Reepithelialization time, wound evaluation, pain	AFS: Average total reepithelialization time was 31.5 days (±24.7) in comparison to 67.9 days (±66.2) in the Jelonet group. AFS: reduced pain levels and infection

Abbreviations: acellular fish skin (AFS); Burn Specific Pain Anxiety Scale (BSPAS); deep partial-thickness burns (DPTB); full thickness (FT); superficial partial-thickness burns (SPTB); total body surface area (TBSA); Visual Analogue Scale (VAS).

## Data Availability

Not applicable.

## References

[1] Oryan A., Alemzadeh E., Moshiri A. (2017). Burn wound healing: Present concepts, treatment strategies and future directions. J. Wound Care.

[2] Markiewicz-Gospodarek A., Kozioł M., Tobiasz M., Baj J., Radzikowska-Büchner E., Przekora A. (2022). Burn Wound Healing: Clinical Complications, Medical Care, Treatment, and Dressing Types: The Current State of Knowledge for Clinical Practice. Int. J. Environ. Res. Public Health.

[3] Alam K., Jeffery S.L.A. (2019). Acellular Fish Skin Grafts for Management of Split Thickness Donor Sites and Partial Thickness Burns: A Case Series. Mil. Med..

[4] Ii R.S., Saathoff E.C., Larson D.A., Wall J.T., Wienandt N.A., Magnusson S., Kjartansson H., Natesan S., Christy R.J. (2021). Accelerated Wound Closure of Deep Partial Thickness Burns with Acellular Fish Skin Graft. Int. J. Mol. Sci..

[5] Júnior E.M.L., Filho M.O.D.M., Costa B.A., Fechine F.V., Vale M.L., Diógenes A.K.D.L., Neves K.R.T., Uchôa A.M.D.N., Soares M.F.A.D.N., de Moraes M.E.A. (2021). Nile Tilapia Fish Skin–Based Wound Dressing Improves Pain and Treatment-Related Costs of Superficial Partial-Thickness Burns: A Phase III Randomized Controlled Trial. Plast. Reconstr. Surg..

[6] Nischwitz S.P., Luze H., Popp D., Winter R., Draschl A., Schellnegger M., Kargl L., Rappl T., Giretzlehner M., Kamolz L.P. (2021). Global burn care and the ideal burn dressing reloaded—A survey of global experts. Burns.

[7] Michael S., Winters C., Khan M. (2019). Acellular Fish Skin Graft Use for Diabetic Lower Extremity Wound Healing: A Retrospective Study of 58 Ulcerations and a Literature Review. Wounds A Compend. Clin. Res. Pract..

[8] Verde M.E.Q.L., Ferreira-Júnior A.E.C., de Barros-Silva P.G., Miguel E.D.C., Mathor M.B., Lima-Júnior E.M., de Moraes-Filho M.O., Alves A.P.N.N. (2021). Nile tilapia skin (*Oreochromis niloticus*) for burn treatment: Ultrastructural analysis and quantitative assessment of collagen. Acta Histochem..

[9] Yang C.K., Polanco T.O., Ii J.C.L. (2016). A Prospective, Postmarket, Compassionate Clinical Evaluation of a Novel Acellular Fish-skin Graft Which Contains Omega-3 Fatty Acids for the Closure of Hard-to-heal Lower Extremity Chronic Ulcers. Wounds.

[10] Le Guellec D., Morvan-Dubois G., Sire J.Y. (2004). Skin development in bony fish with particular emphasis on collagen deposition in the dermis of the zebrafish (*Danio rerio*). Int. J. Dev. Biol..

[11] Brown P. (2001). Bovine Spongiform Encephalopathy and Variant Creutzfeldt-Jakob Disease: Background, Evolution, and Current Concerns. Emerg. Infect. Dis..

[12] Kjartansson H., Olafsson I.H., Karason S., Thorisson H., Baldursson B.T., Gunnarsson E., Jorundsson E., Sigurjonsson G.F. (2015). Use of Acellular Fish Skin for Dura Repair in an Ovine Model: A Pilot Study. Open J. Mod. Neurosurg..

[13] Lullove E., Liden B., Winters C., McEneaney P., Raphael A., Ii J.L. (2021). A Multicenter, Blinded, Randomized Controlled Clinical Trial Evaluating the Effect of Omega-3–Rich Fish Skin in the Treatment of Chronic, Nonresponsive Diabetic Foot Ulcers. Wounds A Compend. Clin. Res. Pract..

[14] Tan S.W., Wong J., Kee T., Chai Z.T., Ho Q.Y., Chan M., Chew K.Y., Oh C.C. (2021). Successful treatment of calciphylaxis in a renal transplant recipient with combination of intralesional sodium thiosulphate, intravenous sodium thiosulphate and fish skin graft. Australas. J. Dermatol..

[15] Dardari D., Lequint C., Jugnet A.C., Bénard T., Bouly M., Penfornis A. (2022). Curing Necrotic Angiodermatitis with an Intact Fish Skin Graft in a Patient Living with Diabetes. Medicina.

[16] Ahn K.H., Park E.S. (2021). A rare case report of neonatal calcinosis cutis induced by distant and delayed extravasation of intravenous calcium gluconate. Arch. Plast. Surg..

[17] Dias M.T.P.M., Bilhar A.P.M., Rios L.C., Costa B.A., Júnior E.M.L., Alves A.P.N.N., Bruno Z.V., Filho M.O.D.M., Bezerra L.R.P.S. (2019). Neovaginoplasty Using Nile Tilapia Fish Skin as a New Biologic Graft in Patients with Mayer-Rokitansky-Küster-Hauser Syndrome. J. Minim. Invasive Gynecol..

[18] Shi Y., Zhang H., Zhang X., Chen Z., Zhao D., Ma J. (2019). A comparative study of two porous sponge scaffolds prepared by collagen derived from porcine skin and fish scales as burn wound dressings in a rabbit model. Regen. Biomater..

[19] Costa B.A., Júnior E.M.L., Filho M.O.D.M., Fechine F.V., De Moraes M.E.A., Júnior F.R.S., Soares M.F.A.D.N., Rocha M.B.S. (2019). Use of Tilapia Skin as a Xenograft for Pediatric Burn Treatment: A Case Report. J. Burn. Care Res..

[20] Wallner C., Holtermann J., Drysch M., Schmidt S., Reinkemeier F., Wagner J.M., Dadras M., Sogorski A., Houschyar K.S., Becerikli M. (2022). The Use of Intact Fish Skin as a Novel Treatment Method for Deep Dermal Burns Following Enzymatic Debridement: A Retrospective Case-Control Study. Eur. Burn J..

[21] Lima-Junior E.M., Filho M.O.D.M., Costa B.A., Fechine F.V., De Moraes M.E.A., Silva-Junior F.R., Soares M.F.A.D.N., Rocha M.B.S., Leontsinis C.M.P. (2019). Innovative treatment using tilapia skin as a xenograft for partial thickness burns after a gunpowder explosion. J. Surg. Case Rep..

[22] Pan S.C. (2013). Burn blister fluids in the neovascularization stage of burn wound healing: A comparison between superficial and deep partial-thickness burn wounds. Burn. Trauma.

[23] Hu Z., Yang P., Zhou C., Li S., Hong P. (2017). Marine Collagen Peptides from the Skin of Nile Tilapia (*Oreochromis niloticus*): Characterization and Wound Healing Evaluation. Mar. Drugs.

[24] Júnior E.M.L., Filho M.O.D.M., Forte A.J., Costa B.A., Fechine F.V., Alves A.P.N.N., De Moraes M.E.A., Rocha M.B.S., Júnior F.R.S., Soares M.F.A.D.N. (2019). Pediatric Burn Treatment Using Tilapia Skin as a Xenograft for Superficial-Partial Thickness Wounds: A Pilot Study. J. Burn. Care Res..

[25] Júnior E.M.L., Filho M.O.D.M., Costa B.A., Rohleder A.V.P., Rocha M.B.S., Fechine F.V., Forte A.J., Alves A.P.N.N., Júnior F.R.S., Martins C.B. (2020). Innovative Burn Treatment Using Tilapia Skin as a Xenograft: A Phase II Randomized Controlled Trial. J. Burn. Care Res..

[26] Reese A.D., Keyloun J.W., Garg G., McLawhorn M.M., Moffatt L.T., Travis T.E., Johnson L.S., Shupp J.W. (2021). Compounded Cerium Nitrate–Silver Sulfadiazine Cream is Safe and Effective for the Treatment of Burn Wounds: A Burn Center’s 4-Year Experience. J. Burn. Care Res..

[27] Moi A.L., Haugsmyr E., Heisterkamp H. (2016). Long-Term Study Of Health And Quality of Life after Burn Injury. Ann. Burn. Fire Disasters.

[28] Ahuja R.B., Goswami P. (2013). Cost of providing inpatient burn care in a tertiary, teaching, hospital of North India. Burns.

[29] Júnior E.M.L., Filho M.O.D.M., Costa B.A., Alves A.P.N.N., De Moraes M.E.A., Uchôa A.M.D.N., Martins C.B., Bandeira T.D.J.P.G., Rodrigues F.A.R., Paier C.R.K. (2020). Lyophilised tilapia skin as a xenograft for superficial partial thickness burns: A novel preparation and storage technique. J. Wound Care.

[30] Badois N., Bauër P., Cheron M., Hoffmann C., Nicodeme M., Choussy O., Lesnik M., Poitrine F.C., Fromantin I. (2019). Acellular fish skin matrix on thin-skin graft donor sites: A preliminary study. J. Wound Care.

[31] Yoon J., Yoon D., Lee H., Lee J., Jo S., Kym D., Yim H., Hur J., Chun W., Kim G. (2022). Wound healing ability of acellular fish skin and bovine collagen grafts for split-thickness donor sites in burn patients: Characterization of acellular grafts and clinical application. Int. J. Biol. Macromol..

[32] Wallace H.J., Fear M.W., Crowe M.M., Martin L.J., Wood F.M. (2017). Identification of factors predicting scar outcome after burn injury in children: A prospective case-control study. Burn. Trauma.

